# Large Vessel Vasculitis After the Administration of Oxford-AstraZeneca COVID-19 Vaccine

**DOI:** 10.31138/mjr.34.1.97

**Published:** 2023-03-31

**Authors:** Olga K. Katsouli, Vasileios G. Lainis, Gerasimos G. Kapellos, Panayiotis G. Vlachoyiannopoulos

**Affiliations:** 1 Department of Pathophysiology, School of Medicine, National and Kapodistrian University of Athens, and Institute for Autoimmune Systemic and Neurologic Diseases, Athens, Greece,; 2 Haematology Department, Athens Medical Center, Athens, Greece

**Keywords:** vasculitis, COVID-19, vaccination

## Abstract

We report a case of a 52-year-old female with Large Vessel Vasculitis (LVV) after vaccination with Oxford-AstraZeneca COVID-19 vaccine. She was presented with fever, started two weeks after the second dose of vaccine. Laboratory values, revealed elevated inflammatory markers and chronic disease anaemia. All the infectious causes were excluded, and immunology tests were negative. Computed Tomography (CT) demonstrated concentric wall thickening of ascending and descending aorta. Positron Emission Tomography (PET) scan showed increased vascular fluorodeoxyglucose (FDG), compatible with LVV. Within one month of treatment with high dose glucocorticoids and iv cyclophosphamide, laboratory findings normalised, and fever resolved.

## INTRODUCTION

The high rate of transmission of the Coronavirus Disease 2019 (COVID-19) and the lack of effective treatment led to a rapid development of vaccines. By the end of 2020, several vaccines were available for use in many countries, over 40 candidate vaccines were under assessment in clinical trials and over 150 undergoing preclinical studies. To date, the European Medicines Agency (EMA) authorized four COVID-19 vaccines: BNT162b2 vaccine (Pfizer and BioNTech, Comirnaty®), mRNA-1273 vaccine (Moderna Therapeutics, SpikeVax®), ChAdOx1 nCoV-19 (AstraZeneca and Oxford University, Vaxzevria®) and Ad26. COV2.S (Janssen COVID19 vaccine, Jcovden®). Although these vaccines are the most powerful weapon against COVID-19 disease and are generally well-tolerated, various adverse events have been observed and reported, including several types of vasculitis.

Herein, we report a case of Large Vessel Vasculitis in a 52-year-old female, 2 weeks after the administration of Oxford-AstraZeneca COVID-19 vaccine (Vaxzevria®).

## CASE PRESENTATION

A previously healthy 52-year-old female presented in our Rheumatology Department with evening fever up to 38.2°C, accompanied by fatigue, that started 5 months ago. The symptoms began two weeks, after the administration of the second dose of AstraZeneca vaccine. Previously, the patient had visited several doctors, who after a rapid work-up to rule out common infections, gave unsuccessful treatments with non-steroid anti-inflammatory drugs.

On physical examination, she was alert, awake, but she was ill-appearing. Her vital signs were within normal limits and the rest of examination was unremarkable. In particular, there was no tenderness upon palpation of the temporal region, arteries were evenly palpated on all extremities and the auscultation of the subclavian and axillary regions was normal.

Complete blood count showed mild anaemia (haemoglobin 10.3 g/dL) with normal differential, ESR=111 mm/hr (ref. range: < 20 mm/hr), C-reactive protein=104 mg/L (ref. range: 0 to 7 mg/L), ferritin levels 560 ng/mL (ref. range: 24 to 307 ng/mL) and plasma fibrinogen levels 773 mg/d (ref. range: 200 to 400 mg/dL). Blood biochemistry was within normal limits.

Viral hepatitis panel, blood and urine cultures, ELISA test for HIV, Wright test, VDRL, serology for EBV, CMV, Quantiferon-TB and PCR test for SARS-CoV2 were all negative. Furthermore, immunology tests including antinuclear antibodies (ANA), antibodies to extractable nuclear antigens (ENAs), anti-mitochondrial (AMA), anti-phospholipid (aPL), and anti-neutrophil cytoplasmic antibodies (ANCAs) were negative. IgG4 serum levels were also normal.

**Figure 1.**
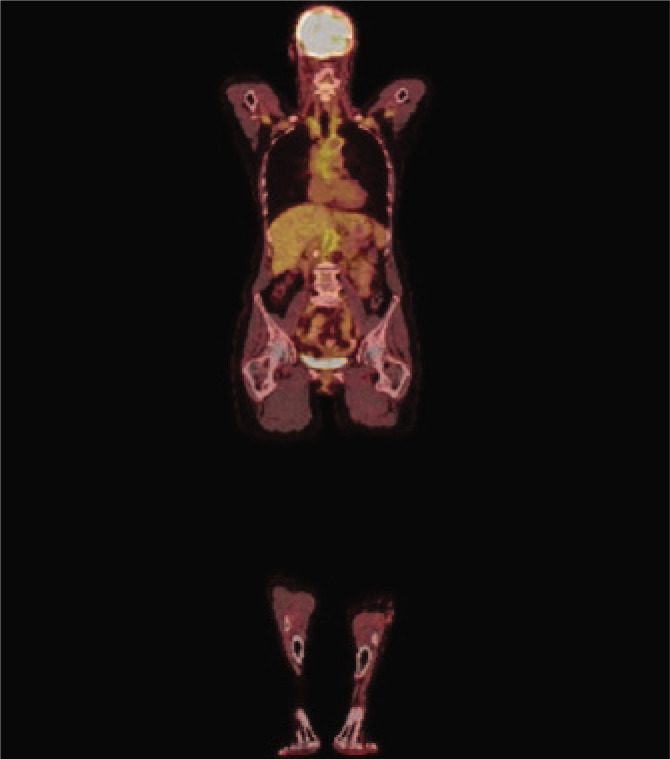


**Figure 2.**
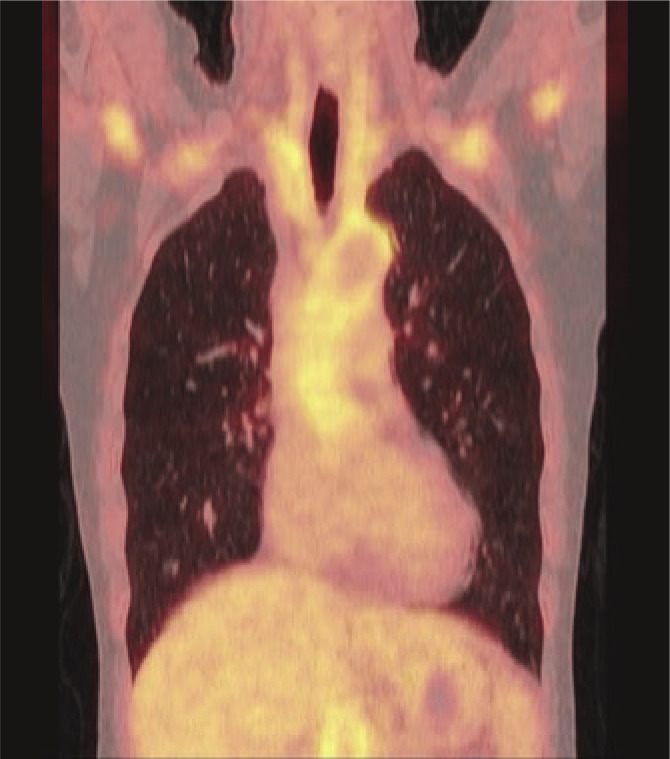


Heart ultrasound revealed mild pericardial effusion with no evidence of valvular vegetations, intramural thrombi, or solid tumours. Contrast-enhanced Computed Tomography (CT) of thorax/abdomen, demonstrated concentric wall thickening of ascending aorta, aortic arch, and descending aorta. Due to these findings, a Positron Emission Tomography (PET) scan was performed, which showed increased fluorodeoxyglucose (FDG) uptake throughout aortic arch, thoracic and abdominal aorta, subclavian, carotid, and axillary arteries, compatible with LVV (**Figure 1, Figure 2**). She was diagnosed as LVV related to vaccination.

According to the 2018 update of the EULAR recommendations for the management of LVV,^1^ we administered to the patient high doses of glucocorticoids (50 mg/day prednisone-equivalent). Due to the extensive vascular damage and the need for approval of tocilizumab administration for LVV treatment by the Hellenic Food and Drug Organization lasting 4 to 8 weeks, the patient was also placed on induction therapy with 6 monthly intravenous pulses of 1g cyclophosphamide, followed by maintenance treatment with subcutaneous tocilizumab 162 mg weekly. The patient did not present any complication during the induction and maintenance treatment.

Within the first month, fever resolved, and the inflammatory markers normalised. Eight months later, the patient remains asymptomatic, receiving treatment with Tocilizumab 162 mg weekly, while the tapering of glucocorticoids is still ongoing.

## DISCUSSION

LVV is defined as the inflammation of the aorta and its major branches. The clinical presentation presents a wide range of symptoms, ranging from general symptoms to potentially life-threatening conditions, such as aortic rupture. The symptoms and signs depend on the underlying cause, whereas the non-specific nature of them requires a high index of suspicion by the clinician to make an early diagnosis.^2^

The aetiological classification of LVV includes various non-infectious and infectious causes. Giant cell arteritis (GCA) and Takayasu’s arteritis are the most common representatives of non-infectious causes, although LVV is also associated with other rheumatic diseases.^3^

As far as the infectious LVV is concerned, the colonisation of damaged endothelium due to haematogenous dissemination of microbes leads to activation of collagenolytic and elastolytic enzymes, resulting in aneurysm formation within weeks of infection. Among the most prevalent causative pathogens are *Streptococcus pyogenes*, *Streptococcus pneumoniae*, *Salmonella* and *Staphylococcus species*.^3^

Vasculitis can be also triggered by some drugs. Several types of vasculitic disorders have been reported after the administration of various vaccines. The dominant responsible vaccine is the influenza vaccine.^4^ Nowadays, several cases of vasculitis have been described in the literature,^5–8^ after both COVID-19 disease or SARS-CoV-2 vaccination (**Table 1**).

**Table 1.** Cases of vasculitis after SARS-CoV-2 vaccination, which have been described in the literature.

**Author year**	**Age/Gender**	**Type of vasculitis**	**Time of onset (days)**	**Dose**	**Type of vaccine**
Hines et al., 2021	40/F	IgA Vasculitis	20	2nd	Pfizer-BioNTech BNT16B2b2
Sirufo et al., 2021	76/F	IgA Vasculitis	7	1st	Oxford-AstraZeneca ChaAdOx1 nCoV-19
Naitlho et al., 2021	62/M	IgA Vasculitis	8	1st	Oxford-AstraZeneca ChaAdOx1 nCoV-19
Vassallo et al., 2021	51/F	Lymphocytic vasculitis	7	1st	Pfizer-BioNTech BNT16B2b2
Kharkar et al., 2021	31/F	Lymphocytic vasculitis	1	2nd	Inactivated viral vaccine COVAXIN®
Ungari et al., 2021	64/M	Lymphocytic vasculitis	1	2nd	Oxford-AstraZeneca ChaAdOx1 nCoV-19
Badier et al., 2021	72/M	IgA Vasculitis	3	1st	Oxford-AstraZeneca ChaAdOx1 nCoV-19
Bostan et al., 2021	33/M	Leukocytoclastic vasculitis	15	1st	Inactivated COVID-19 vaccine (CoronaVac)
Maye et al., 2021	23/F	IgA Vasculitis	3	2nd	Pfizer-BioNTech BNT16B2b2
Obeid et al., 2021	78/F	IgA Vasculitis	2	1st	Moderna mRNA-1273 vaccine
Grossmanet al., 2021	94/M	IgA Vasculitis	7	2nd	Moderna mRNA-1273 vaccine
Iwata et al., 2021	70/F	IgA Vasculitis	10	2nd	Pfizer-BioNTech BNT16B2b2
HakroushandTampe., 2021	79/F	ANCA-associated vasculitis	2	2nd	Pfizer-BioNTech BNT16B2b2
Okuda et al., 2021	37/F	ANCA-associated vasculitis	14	1st	Pfizer-BioNTech BNT16B2b2
Fritzen et al., 2021	60/F	Leukocytoclastic vasculitis	12	2nd	Oxford-AstraZeneca ChaAdOx1 nCoV-19
Cohen et al., 2021	46/F	Leukocytoclastic vasculitis	13	2nd	Pfizer-BioNTech BNT16B2b2
Ball-Burack et al., 2021	22/M	Leukocytoclastic vasculitis	2	1st	Johnson & Johnson SARS-CoV-2 vaccine
Nastroet al., 2021	84/M	Leukocytoclastic vasculitis	2	1st	Pfizer-BioNTech BNT16B2b2
Sandhu et al., 2021	55/F	Leukocytoclastic vasculitis	5	1st	Oxford-AstraZeneca ChaAdOx1 nCoV-19
Sandhu et al., 2021	48/M	Leukocytoclastic vasculitis	5	1st	Oxford-AstraZeneca ChaAdOx1 nCoV-19
Bostan et al., 2021	57/F	Leukocytoclastic vasculitis	7	1st	Pfizer-BioNTech BNT16B2b2
Dickset al., 2021	65/M	Leukocytoclastic vasculitis	2	3rd	Pfizer-BioNTech BNT16B2b2
Bencharattanaphakhi et al., 2021	23/F	Leukocytoclastic vasculitis	2	1st	Inactivated COVID-19 vaccine (CoronaVac)
Bencharattanaphakhi et al., 2021	26/F	Leukocytoclastic vasculitis	2	1st	Inactivated COVID-19 vaccine (CoronaVac)
Kar et al., 2021	46/F	Leukocytoclastic vasculitis	5	1st	Inactivated viral vaccine COVAXIN®
Jin et al., 2021	68/F	Leukocytoclastic vasculitis	2	1st	Oxford-AstraZeneca ChaAdOx1 nCoV-19
Cavalliet al., 2021	57/M	Leukocytoclastic vasculitis	6	1st	Oxford-AstraZeneca ChaAdOx1 nCoV-19
Liang et al., 2021	62/F	Leukocytoclastic vasculitis	7	1st	Oxford-AstraZeneca ChaAdOx1 nCoV-19
Guzmán-Pérez et al., 2021	57/F	Leukocytoclastic vasculitis	1	1st	Oxford-AstraZeneca ChaAdOx1 nCoV-19
Shahrigharahkoshanet al., 2021	77/F	Leukocytoclastic vasculitis	10	1st	Pfizer-BioNTech BNT16B2b2
Erler et al., 2021	42/F	Leukocytoclastic vasculitis	4	1st	Pfizer-BioNTech BNT16B2b2
Colia et al., 2021	22/F	Leukocytoclastic vasculitis	7	2nd	Pfizer-BioNTech BNT16B2b2
Dash et al., 2021	27/M	Urticarial vasculitis	7	2nd	Inactivated COVID-19 vaccine (CoronaVac)
Mückeet al., 2021	76/M	Immune Complex Vasculitis	12	2nd	Pfizer-BioNTech BNT16B2b2
Larson et al., 2021	83/F	Leukocytoclastic vasculitis	7	2nd	Pfizer-BioNTech BNT16B2b2
Larson et al., 2021	35/F	Urticarial vasculitis	1	1st	Moderna mRNA-1273 vaccine
Altun et al., 2021	38/M	Leukocytoclastic vasculitis	4	1st	Pfizer-BioNTech BNT16B2b2
Nazzaro et al., 2021	27/F	Urticarial vasculitis	10	1st	Moderna mRNA-1273 vaccine
Abdelmaksoud et al., 2022	17/F	IgA Vasculitis	10	1st	Pfizer-BioNTech BNT16B2b2
Abdelmaksoud et al., 2022	48/M	Leukocytoclastic vasculitis	4	2nd	Pfizer-BioNTech BNT16B2b2
Gilio et al., 2022	63/F	Large-vessel vasculitis	1	1st	Pfizer-BioNTech BNT16B2b2
Our case	52/F	Large-vessel vasculitis	14	2nd	Oxford-AstraZeneca ChaAdOx1 nCoV-19

Abdelmaksoud et al. reported 40 cases with vasculitis post SARS-CoV2 vaccination, the most common types of them were IgA and Leukocytoclastic vasculitis, while 3 cases of lymphocytic vasculitis, 2 cases of ANCA-associated vasculitis, 3 cases of urticarial vasculitis and 1 case of immune complex vasculitis were also observed. Most cases occurred about 6.2 days after vaccination with mRNA vaccines, while 12 cases had received the ChAdOx1 nCoV-19 AstraZeneca vaccine.^9^ Gilio et al. reported a case of LVV in a 63-year-old patient after the first dose of BNT162b2—Pfizer-BioNTech vaccine. The PET scan performed, showed increased vascular fluorodeoxyglucose (FDG) uptake compatible with LVV, with a picture similar to our patient.^10^

In the period from January 6th, 2021, until August 7th, 2021 the Netherlands Pharmacovigilance Centre Lareb received 68 reports of vasculitic events occurred between 0 and 26 days after the administration of COVID-19 vaccines. The vast majority of cases involved vasculitis limited to the skin, but 11 cases of GCA were also reported.^11^ Furthermore, on October 27^th^, 2022, an analysis of all yellow cards with adverse events that occurred after vaccination with AstraZeneca vaccine during the period 4/1/21 to 26/10/22 was published in the UK. The author emphasises that the adverse reactions are not necessarily due to the vaccine, but the reporter suspected that there is a possible causal and temporal association. Sixty-nine cases of GCA and three cases of aortitis, the type of which was not specified, reported in this analysis print.^12^

The pathophysiologic explanation of the association of LVV with the SARS-CoV-2 vaccine has not been fully established, but is based on the following evidence: The vaccine induces Toll Like Receptor (TLR) activation,^13^ which in turn activates the production of IL-6 linked to disease pathogenesis.^14^ In addition, vaccination-induced antibodies against the spike protein of SARS-CoV-2 cross-react with many human proteins and form immune complexes that disrupt the endothelium.^15^ Finally, there is evidence that patients carrying the *HLA-DRB1* gene are more prone to develop LVV or polymyalgia rheumatica after influenza vaccination, suggesting a coordination of genetic makeup and environmental factors in the induction of vasculitis.^16^

LVV is treated by corticosteroids and tocilizumab, as both induction and maintenance therapy.^1^ Regarding the therapeutic option followed in our case, we have to point out that the administration of tocilizumab for LVV needs approval by the Hellenic Food and Drug Administration which takes one to two months. Therefore, we administered cyclophosphamide based on previous reports.^17–19^

## CONCLUSION

We report a case of development of a large vessel vasculitis, 2 weeks after the administration of the second dose of ChAdOx1 nCoV-19, Oxford-AstraZeneca vaccine. Interestingly, the patient exhibited an extensive vascular inflammation, involving the aorta and its major branches, without presenting symptoms of vascular damage, but only general symptoms, such as fever. We are convinced that, this case will guide the clinicians to be more aware of this complication, without discouraging the vaccination program, the value of which is indisputable.
